# Rapid diagnosis of duck Tembusu virus and goose astrovirus with TaqMan-based duplex real-time PCR

**DOI:** 10.3389/fmicb.2023.1146241

**Published:** 2023-03-30

**Authors:** Haiqin Li, Chunhe Wan, Zhangzhang Wang, Jia Tan, Meifang Tan, Yanbing Zeng, Jiangnan Huang, Yu Huang, Qi Su, Zhaofeng Kang, Xiaoquan Guo

**Affiliations:** ^1^Jiangxi Provincial Key Laboratory for Animal Health, College of Animal Science and Technology, Jiangxi Agricultural University, Nanchang, China; ^2^Institute of Animal Husbandry and Veterinary Medicine, Jiangxi Academy of Agricultural Sciences, Nanchang, Jiangxi, China; ^3^Institute of Animal Husbandry and Veterinary Medicine, Fujian Academy of Agricultural Sciences, Fuzhou, Fujian, China; ^4^Xingguo County Agricultural Technology Extension Center, Ganzhou, Jiangxi, China; ^5^College of Veterinary Medicine, Shandong Agricultural University, Taian, Shandong, China

**Keywords:** diagnosis, Tembusu virus, goose astrovirus, TaqMan, duplex qPCR

## Abstract

The mixed infection of duck Tembusu virus (DTMUV) and goose astrovirus (GoAstV) is an important problem that endangers the goose industry. Although quantitative PCR has been widely used in monitoring these two viruses, there is no reliable method to detect them at the same time. In this study, by analyzing the published genomes of DTMUV and goose astrovirus genotype 2 (GoAstV-2) isolated in China, we found that both viruses have high conservation, showing 96.5 to 99.5% identities within different strains of DTMUV and GoAstV, respectively. Subsequently, PCR primers and TaqMan probes were designed to identify DTMUV and GoAstV-2, and different fluorescent reporters were given to two probes for differential diagnosis. Through the optimization and verification, this study finally developed a duplex TaqMan qPCR method that can simultaneously detect the above two viruses. The lower limits of detection were 100 copies/μL and 10 copies/μL for DTMUV and GoAstV-2 under optimal condition. The assay was also highly specific in detecting one or two viruses in various combinations in specimens, and provide tool for clinical diagnosis of mixed infections of viruses in goose.

## Introduction

Virus infection is an important factor affecting the goose breeding industry, leading to great economic losses (Chen et al., 2013). Rapid testing is the primary tool for clinically monitoring viral infections and developing appropriate treatments. Against the most prevalent viral pathogens circulating in goose, like Goose parvovirus (GPV), Newcastle disease virus (NDV), goose herpesvirus (GHV), and goose adenovirus (GAV), several molecular assays have been developed for clinical diagnosis in the past few years by using either genomic detection methods such as PCR or antigen detection methods such as ELISA (Chen et al., 2009; Guo et al., 2009; Wan et al., 2019; Wu et al., 2019; Tomar et al., 2021). Also, several duplex detection assays were developed for rapid screening of multiple pathogens, usually by duplex PCR (Kong et al., 2007; Chen et al., 2013; Wang et al., 2020). These methods have brought great convenience and benefits to the breeding industry. But unfortunately, there is an endless stream of emerging viruses, which urgently calls for more new approaches to the current epidemic.

Since the emergence of the goose astrovirus (GoAstV, genus Avian Astrovirus, family Astrovirus) in 2017, it has quickly swept through many provinces in China, causing a very serious epidemic (Niu et al., 2018; Yang et al., 2018; Zhang et al., 2018; Wei et al., 2020). The goose astrovirus causes gout and death in 4–16-day-old goslings, the infection and mortality rates in goslings and ducklings can reach 80% and 50%, respectively (Fei et al., 2022). It has resulted in serious economic losses to the poultry-breeding industry. Up to now, several diagnosis tools have been well developed to detect this virus, including immunochromatographic strip assay (Yang et al., 2021), TaqMan-based real-time RT-PCR (Wan et al., 2019; Yin et al., 2020), and loop-mediated isothermal amplification assay (He et al., 2020). However, no work has been done to add this emerging virus to duplex detection systems. Besides, in recent years, the prevalence of duck Tembusu virus (DTMUV, genus Flavivirus, family Flaviviridae) in geese has been increasing (He et al., 2017; Zhu et al., 2022), it mainly causes a sudden laying drop in breeding geese and neurological symptoms in goslings. and it has become the focus of daily pathogen monitoring in geese. Therefore, there is an urgent need to develop rapid screening tools for DTMUV and GoAstV.

In this study, we analyzed the published reference strains of duck Tembusu virus and goose astrovirus, designed specific primers and probes, carried out systematic optimization and verification, and finally established a duplex real-time PCR assay that can accurately detect these two pathogens. The method provides a highly sensitive and accurate detection tool for daily pathogen monitoring in poultry industry.

## Materials and methods

### Viral strains and clinical samples

DTMUV (GenBank: KX977553.1) and GoAstV (genotype 2, GenBank: MZ576222.1) strains used in this study were isolated from China and kept in our laboratory. Goose polyomavirus (GPoV), goose parvovirus (GPV), avian influenza virus (AIV, H5N6), Newcastle disease virus (NDV), avian orthoreovirus (ARV), and avian leukosis virus (ALV) isolated from duck by our laboratory were used to assess specificity of test. Eight DTMUV-positive samples and eight GoAstV-2-positive samples (liver, determined by RT-PCR and sequencing before) were collected from Jan-2022 to July-2022 in our lab. Tissue suspensions (30 μL) were randomly paired to form eight mixed samples. These clinical samples were negative for GPoV, GPV, AIV, NDV, ARV, and ALV through PCR tests.

### Viral DNA/RNA extraction and cDNA synthesis

Viral RNA was extracted by FastPure®Cell/Tissue Total RNA Isolation Mini Kit (Vazyme, Nanjing, China) and then reversely transcripted into cDNA as template using TaKaRa PrimeScript™ RT Master Mix (Takara, Dalian, China). DNA was extracted by TIANamp Genomic DNA Kit (TIANGEN biotech, Beijing, China).

### Design of DTMUV and GoAstV specific qRT-PCR primers and probes

To design appropriate primers and probes against DTMUV and GoAstV, a total of genome sequencing of 89 Tembusu virus strains and 100 goose astrovirus strains published from 2012 to 2022 were downloaded from GenBank as references (Supplementary Table 1). Oligo 7.0 software (Molecular Biology Insights, Inc., Cascade, CO) was used to design specific primers and TaqMan probe against duck Tembusu virus and goose astrovirus, respectively. All primers and probes were synthesized by Shanghai Shengong Life Technology (Shanghai, China). In order to realize the differential diagnosis of duck Tembusu virus and goose astrovirus, two different fluorescent reporters were employed for different probes, namely FAM for DTMUV probe and CY3 for GoAstV probe. Details of these primers and probes can be found in Table 1.

**Table 1:** Primers and probe used in this study.

Virus (accession no.)	Name	Sequence (5′-3′)	Strand	Target gene	Position	Amplicon size (bp)
DTMUV (GenBank: KX977553.1)	Primer-F	CATTGGGAGGACCGAAGAGT	Forward	Polyprotein	3,195	168
	Primer-R	TTGTTGTCCTTGCTGAAGGC	Reverse		3,362	
	Probe	6-FAM-CTGTCGCGGCACGAGCTCGT-DBQ1	Reverse		3,339	
GoAstV (GenBank: MZ576222.1)	Primer-F	CTGCACAAGTTGGTTGGACA	Forward	ORF1ab polyprotein	3,959	111
	Primer-R	CATCATAACGCGTCCAGTCC	Reverse		4,096	
	Probe	Cy3-ACCTGTCACCACCACCAATGAGCC-DBQ1	Reverse		4,036	

### Preparation of an RNA standard by *in vitro* transcription

The target genome fragment of DTMUV and GoAstV were amplified using our designed primers, purified by GenElute™ PCR Purification Kit (Sigma-Aldrich, USA), and then cloned into the pGEM®-T easy Vector system (Promega, Madison, WI, USA) following the instruction and then verified by sequencing in Shanghai Shengong Life Technology (Shanghai, China). The recombinant plasmid was linearized by EcoRI (Promega, Madison, WI, USA) and extracted by phenol: chloroform: isoamyl alcohol. The purified linear DNA was *in vitro* transcribed to RNA using the Riboprobe *in vitro* Transcription System with T7 RNA Polymerase (Promega, Madison, WI, USA) following the manufacturer’s instructions. The transcribed RNA was purified by Trizol (Takara), and then the concentration was quantitated by the DeNovix DS-11 Spectrophotometer (260 nm) and calculated in copy numbers using the following formula. The RNA was stored at −80°C and then used as standard for further analysis.

X copies/μL = [6.02×10^23^ (copies/mol) * RNA concentration (g/μL)]/average Molecular Weight of a base (g/mol) * template length.

### Optimization of duplex TaqMan-based qRT-PCR assay

The qRT-PCR assay was developed using the LightCycler (Roche Diagnostics) and AceQ qPCR Probe Master Mix (Q112-02, Nanjing, China). The concentration of primers or probes and annealing temperature was optimized to ensure specific amplification for the lowest threshold cycle (Ct) value with high fluorescence intensity. The optimum concentrations were selected according to the results of the assay under different formula from 0.1 to 1 μM. Different annealing temperatures were assessed from 58 to 62°C at the interval of 0.5. The optimum temperature was selected based on the measurement results.

### Standard curve

RNA standard was diluted 10-fold serially and amplified with the optimized qRT-PCR system at the concentration of 1.0 × 10^8^–1.0 × 10^1^ copies/μL. Final standard curve is generated based on the CT value and the logarithm of standard copy number.

### Specificity analysis of the duplex TaqMan-based qRT-PCR assay

The nucleic acid of DTMUV, GoAstV-2, and other common viruses, including GPoV, GPV, AIV, NDV, ARV, and ALV, and distilled water were tested to evaluate the specificity of the duplex qPCR test for DTMUV and GoAstV-2 using the abovementioned procedure and parameters. DTMUV and GoAstV-2 were used as positive controls, while distilled water was used as negative controls.

### Sensitivity and reproducibility analysis of the duplex TaqMan-based qRT-PCR assay

RNA standard was diluted 10-fold serially with concentrations ranging from 1.0 × 10^8^–1.0 × 10^1^copies/μL to determine the sensitivity of the assay. Distilled water was tested as a negative control.

To validate the reproducibility of our method, six 10-fold gradient dilutions (1.0 × 10^6^–1.0 × 10^1^ copies/μL) of RNA standard were carried out five times using the abovementioned procedure and parameters, and the standard deviations values of the intra- and inter-assay were calculated.

### Detection of DTMUV and GoAstV in clinical samples using duplex TaqMan-based real-time PCR assay

To further verify the clinical significance of this duplex qPCR assay, we tested the detection ability of this methods in 8 DTMUV-positive samples, 8 GoAstV-positive samples (determined by RT-PCR before), and 8 mixed samples.

### Statistical analysis and visualization

Statistical analyses for standard curve were carried out using SPSS statistic (v1.7.15), and visualization of qPCR amplification curves was done by ABI 7500 software (version 2.3).

## Results

### Genome analysis for priming and probing

Within the downloaded reference strains, both DTMUV and GoAstV exhibited high identities among individual strains from 97.1 to 99.2% (DTMUV) and from 96.5 to 99.5% (GoAstV). Also, the genome distance between these two viruses is very large and stable, with only 45.6–51.2% identities. Sequence analyses using Oligo7 identified two pairs of virus-specific primers and two probes against DTMUV and GoAstV, respectively (Table 1), while both of them show very low identities to another virus.

### Optimization of qRT-PCR system

According to the results of Orthogonal test, primers and probe of 0.2 μM were the most efficient, and the optimized 20 μL qPCR system contains AceQ qPCR Probe Master Mix (10 μL), primers (0.4*2 μL), TaqMan probes (0.2 μL), 50*ROX reference Dye 1 (0.4 μL), cDNA template (1 μL), and ddH_2_O (7.6 μL). After a predenaturation at 95°C for 5 min, the qPCR process includes a predenaturation at 95°C for 5 min, followed by 40 cycles at 95°C for 5 s and at 60°C for 30 s, and ending at 4°C. Fluorescence signals for each sample were harvested at the end of each step at 60°C.

### Standard curve of duplex TaqMan-based qRT-PCR assay

**Figure 1:**
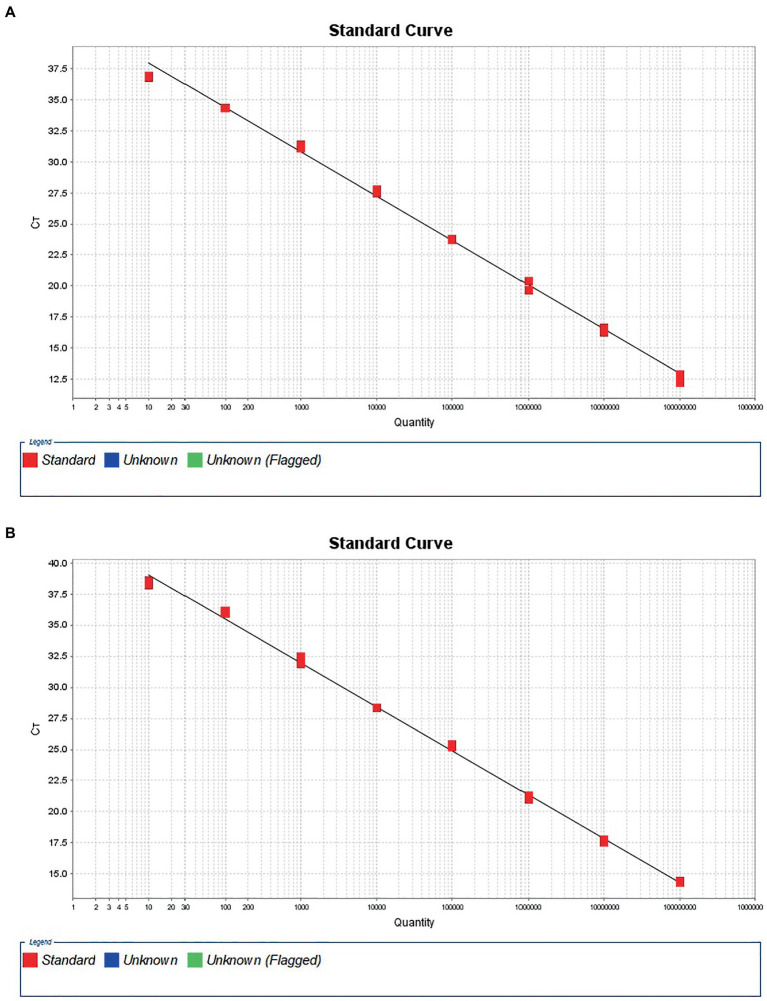
Standard amplification curves for Tembusu virus **(A)** and goose Astrovirus **(B)**.

To construct a standard curve with the logarithm of RNA copy number and the measured Ct values, serial RNA dilutions at the concentration of 1.0 × 10^8^ to 1.0 × 10^1^ copies/μL were tested. Three replicates were tested for each dilution. The optimal curve was selected as the standard curve. Establishment of DTMUV standard curve with abscissa as logarithm of copy number and ordinate as CT value, the correlation coefficient (R^2^) was 0.9986, the slope was −3.588, and the intercept was 41.601. The standard formula for DTMUV is y = −3.588x + 41.60 (Figure 1A, y is Ct value and x is quantity of standard solutions). Establishment of GoAstV standard curve with abscissa as logarithm of copy number and ordinate as CT value, the correlation coefficient (*R*^2^) was 0.998, the slope was −3.533, and the intercept was 42.575 (Figure 1B). The standard formula for GoAstV is y = −3.533x + 42.575. Then, we combined the two viruses template into mixed dilutions for test the inference between them. The results showed a similar standard curve in these mixed samples, revealing no apparent interference between the two primers and probes (Table 2).

**Table 2:** Characterization parameters of standard curves for duplex TaqMan qPCR.

Detection type	Gene	Slope	Y-Inter	*R*^2^	Eff
Single	DTMUV	−3.588	41.601	0.994	90.00%
Single	GoAstV	−3.533	42.575	0.998	91.88%
Duplex	DTMUV	−3.64	40.319	0.997	88.26%
Duplex	GoAstV	−3.515	40.121	0.993	92.521

### Sensitivity of the duplex TaqMan-based qRT-PCR assay

**Figure 2:**
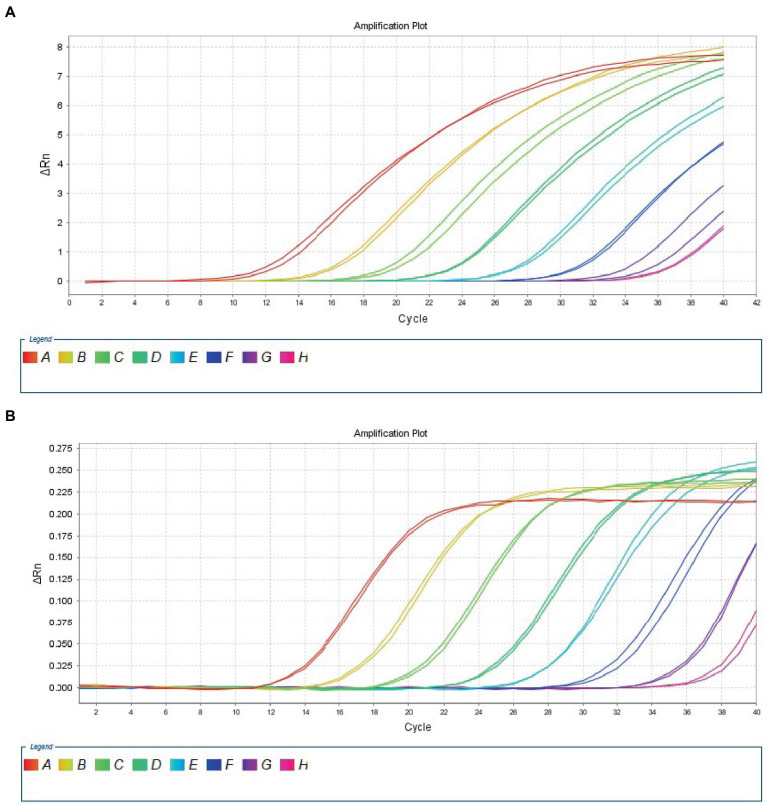
Sensitivity analysis of duplex TaqMan qPCR method for detection of Tembusu virus **(A)** and goose astrovirus **(B)** in unmixed positive standard control. A to H represents standard template at 1.0 × 10^8^–1.0 × 10^1^ copies/μL.

**Figure 3:**
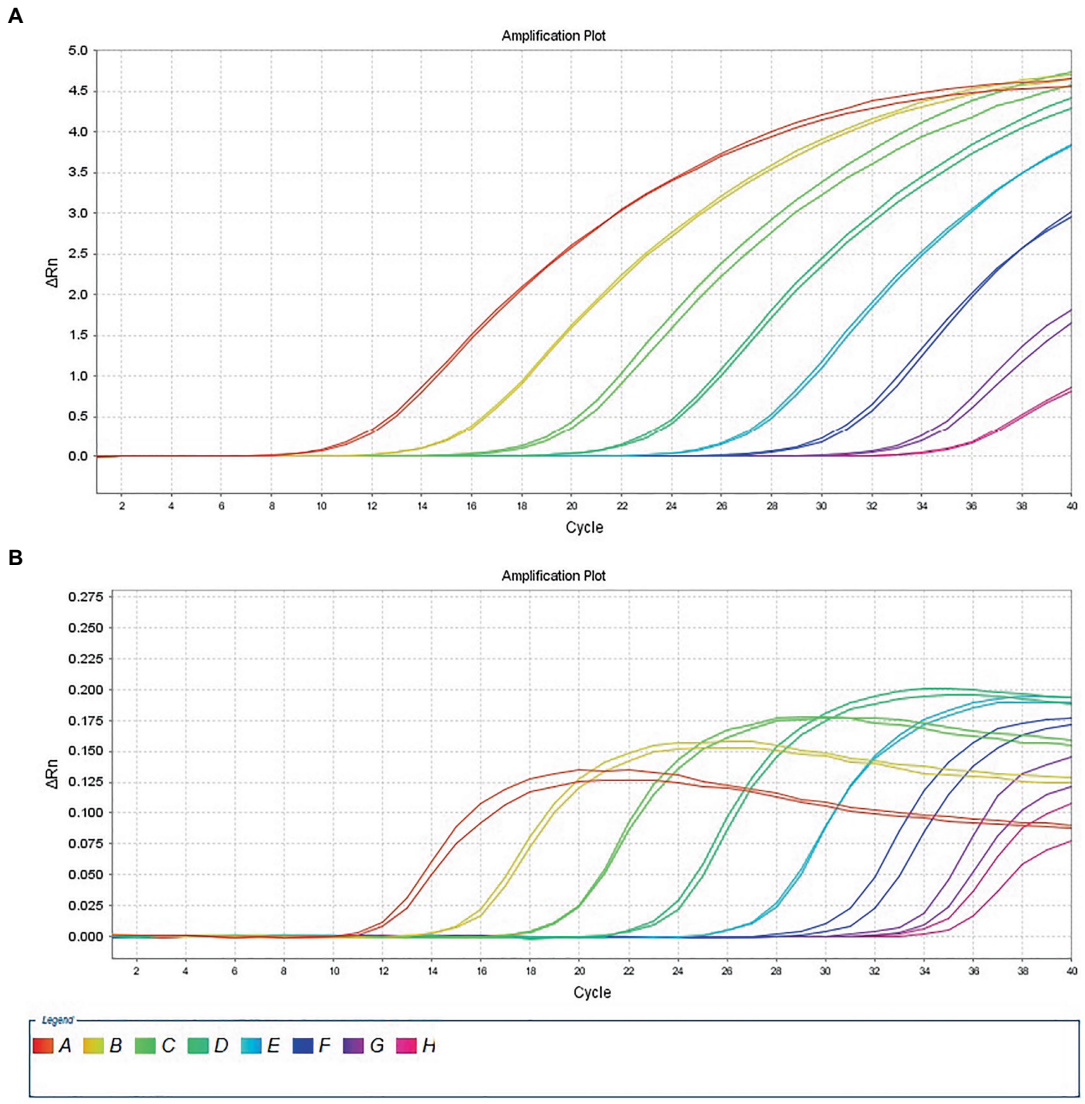
Sensitivity analysis of duplex TaqMan qPCR method for detection of Tembusu virus **(A)** and goose astrovirus **(B)** in mixed positive standard control. A to H represents standard template at 1.0 × 10^8^–1.0 × 10^1^ copies/μL.

The 10-fold gradient dilution of the RNA standard was simultaneously detected by the established qRT-PCR assays (single test). The minimum detection template concentration of the established qPCR was 100 copies/μL and 10 copies/μL, respectively, for DTMUV and GoAstV (Figures 2A,B), indicating that the qRT-PCR assays were highly sensitive. Then, we repeated the above experiments on mixed samples, which yielded similar results (Figures 3A,B), which further confirmed the compatibility of the two detection systems.

### Specificity of the duplex TaqMan-based qRT-PCR assay

DTMUV and GoAstV strains from our laboratory were employed as positive control, and strong fluorescent signals were obtained from reactions. However, for negative control (distilled water used in PCR reaction) and other unrelated avian viruses, the assay gave no signal amplification, confirmed a high specificity of our methods.

### Reproducibility of the duplex TaqMan-based qRT-PCR assay

Next, we tested the reproducibility of our duplex qPCR method at different template concentrations, and the results revealed acceptable standard deviations for both DTMUV and GoAstV-2, ranging from 0.015 to 0.128 or 0.023 to 0.114, respectively, suggesting a high reproducibility for it.

### Detection of DTMUV and GoAstV in clinical mixed RT-PCR-positive samples by duplex TaqMan-based qRT-PCR assay

**Figure 4:**
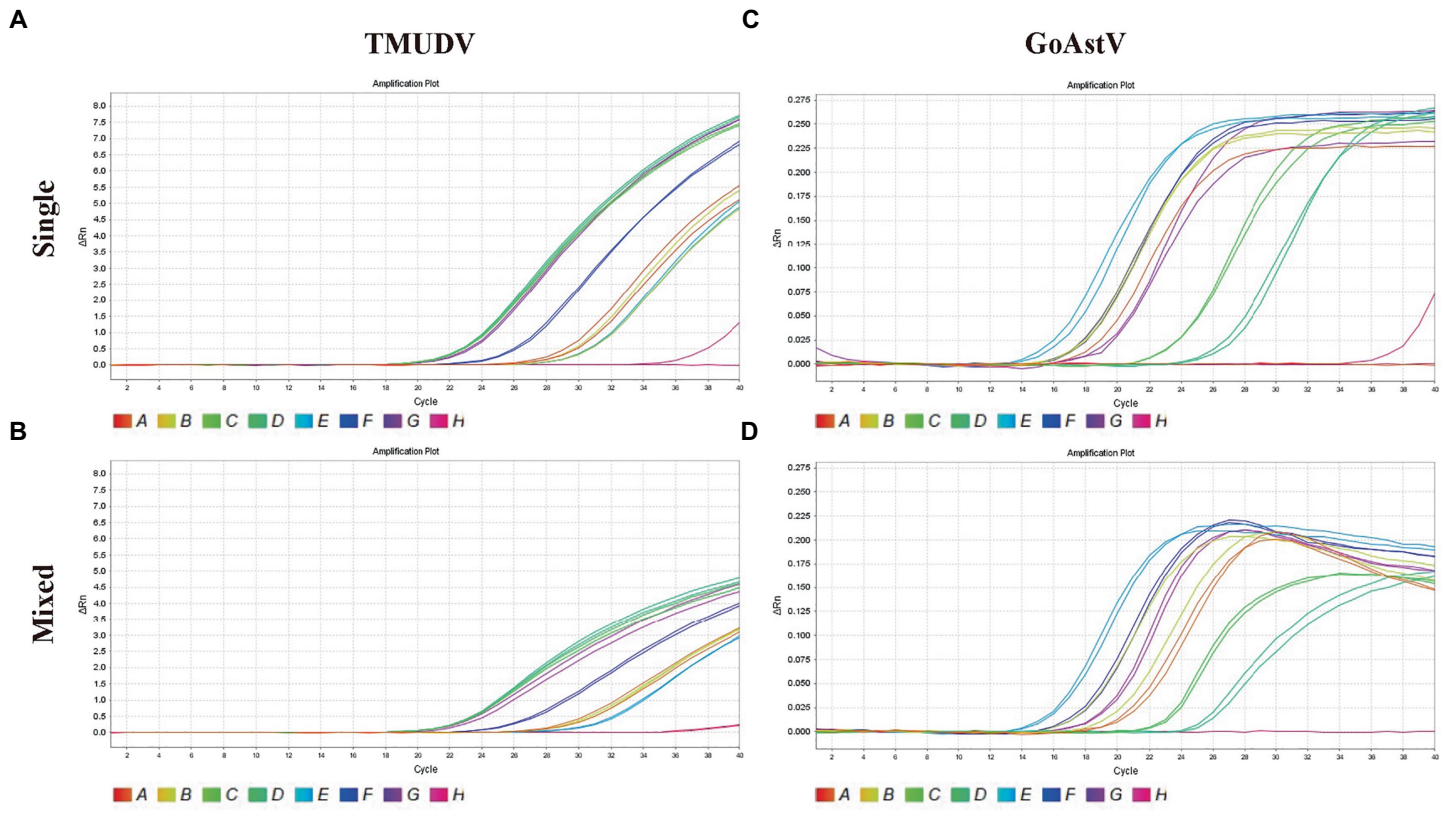
Application of duplex TaqMan qPCR for detection of Tembusu virus (**A**, single positive samples; **B**, mixed positive samples) and goose astrovirus (**C**, single positive samples; **D**, mixed positive samples) in clinical mixed RT-PCR-positive samples.

As shown in Figure 4, the results showed that our methods could both efficiently recognize these two viruses no matter in original samples (single infection) or mixed samples (mixed infection) with consistent results.

## Discussion

The current epidemic of DTMUV and GoAstV has affected most provinces of China, and has no downward tendency (Zhang et al., 2018; Zhu et al., 2022). Although some clinical symptoms can be regarded as robust markers for disease diagnosis, such as joint swellings and typical precipitation of urate for GoAstV infection or neurological symptoms for DTMUV (Niu et al., 2018; Yang et al., 2023), highly sufficient molecular detection methods are needed for early diagnosis. According to our unpublished data, the co-infection of them is highly prevalent, thus a duplex detection system for DTMUV and GoAstV will be of great clinical value in daily pathogen monitoring of poultry industry.

In this study, we firstly analyzed the most recent published genome of DTMUV and GoAstV. These results were highly consistent with previous reports (Niu et al., 2018; Zhang et al., 2018; Qiu et al., 2021; Zhu et al., 2022), suggesting that both of them are comparably stable in genome and there are many conserved regions in DTMUV and GoAstV. Also, the identities between DTMUV and GoAstV were relatively low, indicating that molecular detection methods will be capable of distinguishing them accurately.

Then, we designed two pairs of primers plus virus-specific probes using the Oligo7. After sufficient optimization, we established a duplex diagnostic system with high sensitivity and specificity, which can stably identify DTMUV and GoAstV. More importantly, we used two different fluorescent reporters, namely FAM for DTMUV probe and CY3 for GoAstV probe, thereby we can choose different fluorescent colors to distinguish two viruses in one reaction, which greatly improves detection efficiency and reduces costs. As far as we know, this is the first method that can achieve this differential diagnosis in one reaction, so it will help more breeding enterprises to establish a detection system based on this as soon as possible.

Overall, we reported the first duplex detection system for DTMUV and GoAstV with high sensitivity and specificity. This assay provides a useful tool for studying molecular epidemiology of DTMUV and GoAstV infections in poultry industry, and deserves a board application.

## Data availability statement

The original contributions presented in the study are included in the article/Supplementary material, further inquiries can be directed to the corresponding authors.

## Ethics statement

The animal study was reviewed and approved by the Institutional Animal Care and Use Committee of Jiangxi Agricultural University (Approval ID: JXAULL-2017003). The study protocol and all animal experiments were approved by the Animal Ethics Committee of the Institute of Animal Husbandry and Veterinary, Jiangxi Academy of Agricultural Science (2010-JXAAS-XM-01).

## Author contributions

HL developed the detection method and drafted the manuscript. CW modified the English content of this manuscript. ZK and XG conceived the idea. CW, JT, MT, YZ, and JH validated the performance of diagnostic method. CW, YH, and ZW collected and treated the clinical samples. QS provided important comments on the data analysis and manuscript writing. All authors contributed to the article and approved the submitted version.

## Funding

This work was supported by program for basic research and talent training of the Jiangxi Academy of Agricultural Sciences (JXSNKYJCRC202205), Jiangxi Agriculture Research System (JXARS-09), Fujian Provincial Key Laboratory for Avian Diseases Control and Prevention (FKADL-2022-03), National Natural Science Foundation of China (32072935), the Innovation Team of Jiangxi Agricultural University (JXAUCXTD006), “Thousands of people plan” project of Jiangxi Province (JXSQ2018102118), and Fujian Science and Technology Project (2020J06029, 2021R1026006).

## Conflict of interest

The authors declare that the research was conducted in the absence of any commercial or financial relationships that could be construed as a potential conflict of interest.

## Publisher’s note

All claims expressed in this article are solely those of the authors and do not necessarily represent those of their affiliated organizations, or those of the publisher, the editors and the reviewers. Any product that may be evaluated in this article, or claim that may be made by its manufacturer, is not guaranteed or endorsed by the publisher.

## Supplementary material

SUPPLEMENTARY TABLE S1 Reference strains used in this study.

## References

[ref1] Chen S. Cheng A. Wang M. Zhu D. Luo Q. Liu F. . (2009). Detection and localization of a goose adenovirus in experimentally infected goslings, using indirect immunofluorescence with paraffin-embedded tissue sections. Avian Pathol. 38, 167–174. doi: 10.1080/03079450902737854, PMID: 19322717

[ref2] Chen Z. Li C. Li G. Yu H. Jiang Y. Yan L. . (2013). Rapid diagnosis of goose viral infections by multiplex PCR. J. Virol. Methods 191, 101–104. doi: 10.1016/j.jviromet.2012.09.027, PMID: 23518397

[ref3] Fei Z. Jiao A. Xu M. Wu J. Wang Y. Yu J. . (2022). Genetic diversity and evolution of goose astrovirus in the east of China. Transbound. Emerg. Dis. 69, e2059–e2072. doi: 10.1111/tbed.14542, PMID: 35384346

[ref4] Guo Y. Cheng A. Wang M. Shen C. Jia R. Chen S. . (2009). Development of TaqMan MGB fluorescent real-time PCR assay for the detection of anatid herpesvirus 1. Virol. J. 6:71. doi: 10.1186/1743-422X-6-71, PMID: 19497115 PMC2696427

[ref5] He Y. Wang A. Chen S. Wu Z. Zhang J. Wang M. . (2017). Differential immune-related gene expression in the spleens of duck Tembusu virus-infected goslings. Vet. Microbiol. 212, 39–47. doi: 10.1016/j.vetmic.2017.08.002, PMID: 29173586

[ref6] He D. Yang J. Jiang X. Lin Y. Chen H. Tang Y. . (2020). A quantitative loop-mediated isothermal amplification assay for detecting a novel goose astrovirus. Poult. Sci. 99, 6586–6592. doi: 10.1016/j.psj.2020.09.077, PMID: 33248574 PMC7705033

[ref7] Kong L.-C. Ren T. Ao Y.-H. Xi R.-Z. Liao M. (2007). Multiplex Rt-PCR for virulence detection and differentiation between Newcastle disease virus and goose-origin APVM-1. Avian Dis. 51, 668–673. doi: 10.1637/0005-2086(2007)51[668:MRFVDA]2.0.CO;2, PMID: 17992924

[ref8] Niu X. Tian J. Yang J. Jiang X. Wang H. Chen H. . (2018). Novel goose astrovirus associated gout in gosling, China. Vet. Microbiol. 220, 53–56. doi: 10.1016/j.vetmic.2018.05.006, PMID: 29885801

[ref9] Qiu G. Cui Y. Li Y. Li Y. Wang Y. (2021). The spread of Tembusu virus in China from 2010 to 2019. Virus Res. 300, 198374–198377. doi: 10.1016/j.virusres.2021.198374, PMID: 33775750

[ref10] Tomar P. Joshi V. G. Mahajan N. K. Jindal N. (2021). Multiple antigenic peptide-based flow through dot-blot assay for simultaneous antibody detection of infectious bronchitis virus and Newcastle disease virus. Biologicals 73, 24–30. doi: 10.1016/j.biologicals.2021.07.005, PMID: 34389244

[ref11] Wan C. Chen C. Cheng L. Fu G. Shi S. Liu R. . (2019). Specific detection of the novel goose astrovirus using a TaqMan real-time RT-PCR technology. Microb. Pathog. 137:103766. doi: 10.1016/j.micpath.2019.103766, PMID: 31580957

[ref12] Wan C. Chen C. Cheng L. Liu R. Shi S. Fu G. . (2019). Specific detection and differentiation of classic goose parvovirus and novel goose parvovirus by TaqMan real-time PCR assay, coupled with host specificity. BMC Vet. Res. 15:389. doi: 10.1186/s12917-019-2090-7, PMID: 31676004 PMC6823957

[ref13] Wang Y. Cui Y. Li Y. Jiang S. Liu H. Wang J. . (2020). Simultaneous detection of duck circovirus and novel goose parvovirus via SYBR green I-based duplex real-time polymerase chain reaction analysis. Mol. Cell. Probes 53:101648. doi: 10.1016/j.mcp.2020.101648, PMID: 32798710 PMC7426261

[ref14] Wei F. Yang J. Wang Y. Chen H. Diao Y. Tang Y. (2020). Isolation and characterization of a duck-origin goose astrovirus in China. Emerg. Microbes. Infect. 9, 1046–1054. doi: 10.1080/22221751.2020.1765704, PMID: 32486971 PMC7448921

[ref15] Wu X. Song Z. Zhai X. Zuo L. Mei X. Xiang R. . (2019). Simultaneous and visual detection of infectious bronchitis virus and Newcastle disease virus by multiple LAMP and lateral flow dipstick. Poult. Sci. 98, 5401–5411. doi: 10.3382/ps/pez372, PMID: 31265112 PMC7107193

[ref16] Yang S. Shi Y. Wu J. Chen Q. (2023). Ultrastructural study of the duck brain infected with duck Tembusu virus. Front. Microbiol. 14, 1–9. doi: 10.3389/fmicb.2023.1086828, PMID: 36891400 PMC9987711

[ref17] Yang J. Tian J. Tang Y. Diao Y. (2018). Isolation and genomic characterization of gosling gout caused by a novel goose astrovirus. Transbound. Emerg. Dis. 65, 1689–1696. doi: 10.1111/tbed.12928, PMID: 29920970

[ref18] Yang X. Wei F. Tang Y. Diao Y. (2021). Development of immunochromatographic strip assay for rapid detection of novel goose astrovirus. J. Virol. Methods 297:114263. doi: 10.1016/j.jviromet.2021.114263, PMID: 34391804

[ref19] Yin D. Yang J. Tian J. He D. Tang Y. Diao Y. (2020). Establishment and application of a TaqMan-based one-step real-time RT-PCR for the detection of novel goose-origin astrovirus. J. Virol. Methods 275:113757. doi: 10.1016/j.jviromet.2019.113757, PMID: 31669331

[ref20] Zhang X. Ren D. Li T. Zhou H. Liu X. Wang X. . (2018). An emerging novel goose astrovirus associated with gosling gout disease, China. Emerg. Microbes Infect. 7:152. doi: 10.1038/s41426-018-0153-7 30185786 PMC6125322

[ref21] Zhu Y. Hu Z. Lv X. Huang R. Gu X. Zhang C. . (2022). A novel Tembusu virus isolated from goslings in China form a new subgenotype 2.1.1. Transbound. Emerg. Dis. 69, 1782–1793. doi: 10.1111/tbed.14155 33993639

